# Precision in prediction: tailoring machine learning models for breast cancer missense variants pathogenicity prediction

**DOI:** 10.1093/bib/bbaf611

**Published:** 2025-11-20

**Authors:** Rahaf M Ahmad, Noura AlDhaheri, Mohd Saberi Mohamad, Bassam R Ali

**Affiliations:** Department of Genetics and Genomics, College of Medicine and Health Sciences, United Arab Emirates University, Al Ain, P.O. Box 15551, Sheikh Khalifa Bin Zayed Street, Al Maqam District, Abu Dhabi Emirate, United Arab Emirates; Department of Genetics and Genomics, College of Medicine and Health Sciences, United Arab Emirates University, Al Ain, P.O. Box 15551, Sheikh Khalifa Bin Zayed Street, Al Maqam District, Abu Dhabi Emirate, United Arab Emirates; Department of Genetics and Genomics, College of Medicine and Health Sciences, United Arab Emirates University, Al Ain, P.O. Box 15551, Sheikh Khalifa Bin Zayed Street, Al Maqam District, Abu Dhabi Emirate, United Arab Emirates; Centre for Advanced Analytics, CoE for Artificial Intelligence, Faculty of Engineering & Technology, Multimedia University, Jalan Ayer Keroh Lama, Melaka, 75450 Bukit Beruang, Malaysia; Department of Biosystems Engineering, Faculty of Agricultural Technology, Universitas Brawijaya, Malang, East Java, Jawa Timur 65145, Indonesia; Institute For Data Innovation and Artificial Intelligence, Cranbourne East, VIC 3977, Australia; Department of Genetics and Genomics, College of Medicine and Health Sciences, United Arab Emirates University, Al Ain, P.O. Box 15551, Sheikh Khalifa Bin Zayed Street, Al Maqam District, Abu Dhabi Emirate, United Arab Emirates

**Keywords:** breast cancer, pathogenicity prediction, machine learning, genetic missense variants

## Abstract

Accurate classification of genetic variants is critical for precision medicine, particularly hereditary diseases such as breast cancer. However, widely used tools like MutPred and Combined Annotation Dependent Depletion (CADD) offer genome-wide pathogenicity predictions that often overlook disease-specific variant behavior, limiting their clinical utility. This study addresses that gap by training and benchmarking nine machine learning (ML) models-including ensemble and baseline classifiers-on a breast cancer gene-specific dataset rich in conservation scores, functional annotations, and allele frequency features. Among all models, the Extra Trees model achieved the highest performance, with an accuracy of 0.999 and a 95% confidence interval of (0.998–1.000). recursive feature elimination identified the most informative genomic features, enhancing model efficiency. To ensure clinical transparency, we applied interpretability techniques including Local Interpretable Model-Agnostic Explanations and permutation feature importance, which highlighted the key drivers of each prediction. The calibration curve further confirmed the reliability of predicted probabilities, supporting their potential use in clinical decision-making. On an independent ClinGen dataset, Extra Trees achieved 99.1% accuracy and outperformed widely used predictors confirming its robustness and clinical applicability. This is the first comprehensive benchmarking study to apply ML models specifically to breast cancer-related missense variants using disease-gene-specific training data and integrated interpretability. Our results show that disease-specific ML approaches outperform general predictors, offering improved reliability, transparency, and relevance to clinical genomics. By bridging the gap between broad genome-wide tools and tailored clinical prediction, this study lays the foundation for implementing ML-driven pathogenicity prediction in breast cancer diagnostics and precision medicine, with potential expansion to other disease contexts.

## Introduction

The rapid advancement of next-generation sequencing (NGS) has led to an exponential increase in the detection of variants, specifically missense variants, uncovering new genes associated with genetic disorders. Variant classification is crucial to precision medicine, playing a critical role in diagnosing and managing hereditary diseases, including breast cancer. However, interpreting these variants is complex, often requiring integration of diverse data sources that can sometimes lead to conflicting conclusions [1]. Genetic variants are categorized based on their impact on gene function and disease risk. They are classified into five tiers following the framework developed by the American College of Medical Genetics and Genomics and the Association for Molecular Pathology (ACMG-AMP). This evidence-based system incorporates data such as population frequency, functional studies, and computational predictions to provide standardized interpretation [1]. Despite the utility of these guidelines, traditional approaches heavily rely on expert judgment and predefined rules. These processes are labor-intensive, subjective, and prone to inconsistencies across laboratories, particularly given the high-dimensionality and complexity of genetic data [2]. Moreover, as whole-genome sequencing and whole-exome sequencing is becoming routine in clinical practice, the sheer volume of variants identified adds significant challenges for manual interpretation. To address these limitations, machine learning (ML) models are emerging as powerful tools for variant classification. By automating the interpretation process, ML approaches offer scalability, consistency, and improved accuracy. They can effectively handle the complexity of genetic datasets, making them a promising solution for enhancing the reliability of variant interpretation in precision medicine [3, 4].

ML has revolutionized genomics by providing scalable solutions for analyzing large datasets and identifying complex patterns that surpass human intuition. Tools like RENOVO [5], Lyrus [6], Combined Annotation Dependent Depletion (CADD) [7], and others have demonstrated the potential of ML in predicting variant pathogenicity. These models integrate a range of predictive features to deliver probabilistic predictions of pathogenicity. However, genome-wide predictors, including CADD and MutPred, do not incorporate disease-specific molecular mechanisms, leading to suboptimal performance for breast cancer-related genes. These tools are designed for broad applicability and rely on generic variant patterns, disregarding the disease-relevant biological context that could enhance accuracy in a clinical setting. The limited transparency of these models raises concern among healthcare providers who need clear justifications for predictions to build trust and confidence in clinical decision-making [1, 8]. Genome-wide ML approaches, which leverage variants across the entire genome to train pathogenicity predictors, are widely used. Tools like MutPred [9], Leap [10], and SIFT [11] are examples of genome-wide predictors that benefit from large number of training variants, reducing variance and improving predictive performance [7, 12]. However, they lack disease specificity, often leading to misclassification of variants in genes with complex disease associations. For instance, the impact of a genetic variant can vary significantly between diseases, such as hereditary cancer syndromes and cardiovascular disorders. Consequently, disease-specific and gene-specific ML models are gaining prominence due to their ability to tailor predictions to the biological context of a specific disease or gene. Disease-specific ML models focus on variants from genes associated with a particular disease or group of related disorders [13–15]. Several studies have demonstrated the superiority of disease-specific models over general genome-wide approaches. For instance, Zhang *et al*. [13] developed CardioBoost, a disease-specific classifier for inherited cardiac conditions, showing significantly higher precision and clinical utility compared to general tools like REVEL and CADD. Similarly, Chen *et al*. [14] introduced DS-MVP, a deep learning framework leveraging disease-specific fine-tuning, which outperformed 20 existing tools in classifying pathogenic missense variants under distinct disease conditions, including cardiomyopathy and neurodegeneration. Furthermore, Kang *et al*. [15] showed that disease-specific models trained on hereditary cancer gene sets including *BRCA1/2* provided improved prediction performance over genome-wide tools and emphasized that such models capture disease-relevant variant features that broader models often overlook. Breast cancer-specific studies have shown that general pathogenicity predictors underestimate the relevance of *BRCA1/BRCA2-*associated variants, leading to inconsistent classification outcomes. Some studies highlighted the superior performance of breast cancer-specific models in interpreting pathogenicity of variants in *BRCA1*, *BRCA2*, and other breast cancer-related genes [16–19]. These models integrate knowledge of hereditary cancer pathways, population-specific allele frequencies, and functional annotations to achieve greater precision in predicting variant pathogenicity. Building on this, gene-specific ML models take a more focused approach by training predictors using variants from a single gene, such as *BRCA1* or *BRCA2*. This specificity allows the models to capture gene-level patterns of pathogenicity, potentially leading to higher accuracy. Some researchers developed gene-specific predictors for *BRCA1* and *BRCA2*, showing their superiority over genome-wide predictors in evaluating breast cancer-associated variants [15, 20]. However, despite their accuracy, these gene-specific models lack generalizability, making them less effective for broader breast cancer pathogenicity prediction. A trade-off exists-while gene-specific models may achieve higher precision for variants in a single gene, disease-specific models encompass a broader context of disease-related genes, offering a balance between specificity and variability. Existing ML-based pathogenicity predictors predominantly focus on high-risk, well-established genes, overlooking the full spectrum of disease-relevant genetic variants in breast cancer. This gap in research limits the potential of ML-based tools to provide comprehensive, clinically relevant predictions. The absence of a breast cancer disease-gene specific pathogenicity prediction tool leaves a critical gap in leveraging the full potential of ML to improve variant classification. Such a tool would enable the integration of breast cancer-specific data to provide more precise and clinically actionable predictions. This study directly addresses this gap by developing and evaluating ML models trained on a curated breast cancer-specific dataset. By systematically comparing multiple models and employing interpretability techniques, we establish a novel framework for disease-specific pathogenicity prediction of breast cancer that can be extended to other genetic diseases as well. Therefore, this study employs several ML models on a gene-disease specific dataset for breast cancer, offering a comprehensive solution that outperforms genome-wide predictors in accuracy and clinical relevance. To our knowledge, this is the first comprehensive benchmarking study with disease-gene-specific training data and advanced interpretability techniques to support clinical-grade pathogenicity prediction.

## Methodology

### Overview of the machine learning workflow

**Figure 1:**
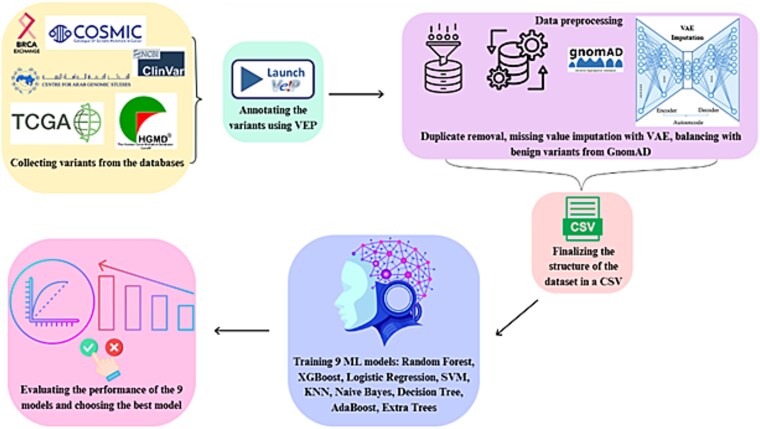
The schematic representation of the research workflow.

In this study, we developed a robust ML pipeline to evaluate and compare multiple algorithms for predicting the pathogenicity of breast cancer genetic missense variants. Our methodology focused on ensuring high-quality preprocessing, rigorous feature selection, robust model evaluation, and transparent result interpretation. Figure 1 provides a schematic representation of the research workflow.

The ML workflow for genetic variant classification follows a structured pipeline, ensuring robust data processing, model training, and interpretability. The process begins with data input, where genomic datasets, typically in Comma Seperated Value (CSV) format, are loaded and prepared for analysis. Next, data preprocessing is performed, involving cleaning, encoding categorical variables, and filtering out irrelevant or redundant features. This is followed by feature selection, where techniques like recursive feature elimination (RFE) and correlation filtering are applied to retain only the most informative models, reducing dimensionality, and enhancing model performance.

Once the data is refined, model training and evaluation take place, wherein multiple models, such as Random Forest, XGBoost, and Support Vector Machines (SVM), are trained and assessed using performance metrics like Area Under the Curve (AUC), precision, recall, and F1-score. To enhance the transparency of model predictions, an interpretability analysis is conducted using techniques like Interpretable Model-Agnostic Explanations (LIME) and SHapley Additive exPlanations (SHAP), providing insights into feature contributions. Following this, statistical validation is performed using methods such as *z*-tests, *f*-tests, and calibration curve to ensure model reliability and significance. Finally, the output and visualization stage consolidate predictions, feature importance rankings, and performance metrics into visual representations, facilitating easy interpretation and application in genomic research, and clinical decision-making. This workflow optimally integrates ML models along with comprehensive disease-gene specific training dataset, interpretability and statistical rigor, providing a reliable framework for variant classification. Below are the details of each step of the methodological framework.

### Data collection

The breast cancer gene panel used in this study was derived through a literature review and a benchmarking study combined with evidence from curated variant repositories [21, 22]. Specifically, high-penetrance susceptibility genes (*BRCA1, BRCA2, TP53, ATM, CHEK2, CDH1*) were prioritized due to their well-documented roles in DNA damage repair, cell-cycle control, and familial breast cancer predisposition. Additional genes were cross validated against ClinVar, COSMIC, TCGA, and BRCAExchange to ensure that selected loci contained clinically annotated missense variants with pathogenicity evidence. This integrative approach ensured that the final panel reflected both biological plausibility and clinical utility, covering key genes implicated in hereditary and sporadic breast cancer. The complete gene list with variant counts is provided in Supplementary Table S1. Eventually, the first step was data collection, where genetic variants were extracted from multiple online databases. Cancer-specific databases included COSMIC, cBioPortal, TCGA, and the Catalog for Transmission Genetics in Arabs, while general databases such as ClinVar and HGMD were also utilized. These databases were chosen based on previous research in which we benchmarked the best datasets in terms of quality-size relation for breast cancer genetic variant pathogenicity prediction using AutoML frameworks [23]. This research indicated that selecting a disease-specific but comprehensive gene list and collecting variants from both cancer-specific and general databases significantly improved model performance. Primarily, a comprehensive list of breast cancer related genes was collected through a literature review (Supplementary Table S1), those genes were used as keywords to look for variants related to breast cancer. The keywords “breast” and “cancer” were used as disease/phenotype filtering criteria, while for the gene name, each gene was individually used. Variants with the classification of Pathogenic or Likely Pathogenic were included in Pathogenic category of the dataset, and variants with the classification of Benign or Likely Benign were included in the Benign category of the dataset. All other classifications including variants of unknown significances were excluded from the dataset. For this study, only missense single nucleotide variants were included. The collected variants were filtered to include only those associated with breast cancer-related phenotypes and breast cancer-specific genes, as highlighted in our previous studies [21, 22]. Annotation of the variants was performed using the Variant Effect Predictor by Ensembl [24], and the data was thoroughly reviewed for accuracy.

### Data preprocessing

Data preprocessing involved removing duplicates, handling missing values, encoding categorical variables, balancing the dataset, and preparing it for downstream analysis. Duplicated values were filtered out using the drop_duplicate function in Python. After filtering duplicates and irrelevant entries, the dataset comprised 5443 pathogenic variants and 5439 benign variants. Missing values were imputed using Variational Autoencoders (VAE) implemented via TensorFlow/Keras. VAEs are generative models that compress high-dimensional data into a lower-dimensional latent space before reconstructing it, making them highly suitable for genetic datasets, which are often sparse and interdependent [25]. To assess imputation strategies, we conducted a benchmarking study of VAE against MICE and K-Nearest Neighbors (KNN). As shown in Supplementary Fig. 1, the comparison of KNN, MICE, and VAE imputers across increasing missingness rates. While MICE achieved the highest AUC and F1 at low missingness, VAE maintains the most stable performance across all metrics, highlighting its robustness under higher data loss. Root Mean Squared Error (RMSE), Mean Absolute Error (MAE), and additional benchmarks are provided in Supplementary Table S2. Categorical variables were label-encoded using the LabelEncoder package to ensure compatibility with ML models. To address class imbalance, we expanded the benign variant set using population allele frequency data from gnomAD, guided by ACMG/AMP population frequency criteria. Variants with high population frequency were considered likely benign, while those reported as pathogenic in ClinVar were excluded. Benign and pathogenic variants were then balanced on a per-gene basis to reduce bias, and cross-validation was stratified by both gene and class. This approach ensured that genes with many variants, such as *BRCA1*, *BRCA2*, and *TP53*, remained highly represented, while all genes contributed proportionally, preserving both fairness and biological relevance in model evaluation. The balancing is summarized in Supplementary Table S3, which shows representative gene-level counts before and after balancing, highlighting the proportional augmentation of benign variants from gnomAD. The final dataset used in the deployment included 5443 pathogenic variants and 5443 benign variants. All preprocessing steps were implemented using Python 3.10 in the PyCharm IDE.

### Feature selection and engineering

To evaluate the role of feature selection, we compared classifier performance with and without reduction, and since increasing features did not substantially improve performance (Supplementary Table S7), we proceeded with feature selection. To reduce multicollinearity, we benchmarked Pearson correlation thresholds (0.70–0.95) and found that |r| > 0.90 provided the best trade-off, yielding 42 features out of 151 with the highest cross-validated AUC (Supplementary Table S4) [26]. Recursive Feature Elimination with Cross-Validation (RFECV) analysis (Supplementary Fig. 2) showed that predictive performance plateaued after around 15–20 features, indicating that only a subset was strictly required for maximal accuracy. Nevertheless, we retained 42 features as the final set (Supplementary Table S5) to balance predictive performance with biological interpretability and to avoid over-pruning. These features span allele frequency, conservation, protein function, splicing, and ensemble pathogenicity scores. Feature importance analyses (RFE, RFECV) confirmed that this streamlined panel captured the strongest biological signals without loss of accuracy, with key contributors including allele frequency metrics, conservation scores, protein function predictors, and splicing predictors.

### Model development and evaluation

A suite of ML algorithms was deployed, encompassing both ensemble and traditional models, including Random Forest, XGBoost, Logistic Regression, SVM, KNN, Naive Bayes, Decision Trees, AdaBoost, and Extra Trees models. The dataset was split into training (80%) and testing (20%) sets, ensuring that model evaluation was conducted on unseen data. For each predictor, a comprehensive set of performance metrics was computed, including AUC, precision, recall, F1 score, sensitivity, specificity, Matthew’s correlation coefficient (MCC), and Cohen’s kappa. To ensure models robustness, 5-fold cross-validation was conducted, further validating the stability and generalizability of each model. All results were systematically documented and saved for subsequent analysis.

To ensure both performance consistency and computational feasibility, we adopted a two-phase evaluation strategy. In the initial phase, models were trained and evaluated using a fixed random seed (Seed 42), allowing for standardized model benchmarking, performance metric generation, and preliminary interpretability assessments. Based on these findings, a second phase was introduced to identify the best-performing seed for each model by evaluating across multiple random seeds and selecting the seed with the highest AUC.

In the second phase, final interpretability analyses—including LIME explanations for four representative samples (TP, TN, FP, FN), permutation feature importance (PMI), and bootstrapped confidence intervals for performance metrics and Receiver Operating Characteristic (ROC) curves, were computed using the best-performing seed for the top-ranked classifier (Extra Trees). This phased approach enabled a balance between initial result reproducibility and final evaluation rigor, while ensuring that all interpretability outputs reflect the most stable and optimal model configuration.

### Multi-seed evaluation and best seed selection

To ensure robust and reproducible model performance, each classifier was evaluated across multiple random seeds (42, 101, 202, 303, 404). For each seed, models were trained and tested using the same stratified 80:20 data split, and performance metrics-particularly AUC-were recorded. This multi-seed evaluation enabled assessment of performance stability and variability due to initialization randomness.

The best-performing seed for each model was selected based on the highest average AUC across metrics. This approach ensured that downstream interpretability analyses (LIME, PMI) and visualizations were derived from the most stable and generalizable configuration of each model.

### Threshold optimization and prediction

To optimize classification performance, ROC and Precision-Recall curves were generated for each model (Figs. 3 and 4), and the optimal thresholds were determined through the ROC curve. These thresholds were applied to classify predictions into clinically relevant categories, such as “Pathogenic” and “Benign”.

### Statistical validation

Statistical tests were conducted to evaluate the significance of differences between model performances. These included *z*-tests, Shapiro–Wilk tests, Levene’s test, and *f*-tests or Analysis of Variance (ANOVA), performed on the predicted probabilities and performance metrics of each model (Fig. 5A and B). This statistical validation ensured that the observed differences were robust and not due to random chance. Results were visualized and annotated with significant levels to provide clear and interpretable insights into model performance.

### Model interpretability and reliability assessment

To enhance the transparency of model decisions, explainability techniques were integrated into the workflow. PMI analysis provided a global perspective on feature relevance across the dataset (Fig. 6A, B, and C). Additionally, LIME were employed to elucidate feature contributions to individual predictions (Fig. 7A, B, and C). Calibration curves were also generated to assess the alignment of predicted probabilities with observed outcomes, ensuring the reliability of model outputs (Fig. 8).

Using TreeExplainer, we computed SHAP values for the best-seed Extra Trees model and summarized global contributions with a beeswarm of the top 20 features. SHAP confirms the permutation-based ranking, with meta-predictors (e.g. ClinPred/BayesDel) and evolutionary constraints (phyloP/phastCons) driving predictions. We further provide LIME-style SHAP waterfall plots for the most confidently pathogenic and benign cases to show directionality and magnitude at the variant level. These plots explain why a specific variant crosses the decision threshold (features pushing right increase pathogenicity; features pushing left decrease it).

### Selection of the best predictor

A weighted scoring system was devised to select the best-performing predictor in addition to other metrics. Metrics were assigned weights based on their clinical relevance, with higher priority given to AUC, precision, and recall. The predictor achieving the highest weighted score was identified as the optimal model, balancing overall performance with clinical applicability. Following model selection based on weighted scores and AUC performance, we conducted an in-depth interpretability analysis specifically on the best-performing model (Extra Trees), using its optimal seed identified through multi-seed evaluation. Local LIME was applied to four representative samples: a true positive (TP), true negative (TN), false positive (FP), and false negative (FN), to examine feature contributions in both accurate and erroneous classifications. This approach allowed for a more granular understanding of the model’s decision-making behavior in clinically relevant scenarios.

In parallel, PMI was re-evaluated for the Extra Trees model using the same optimal seed to ensure consistency and model stability. This analysis quantified the impact of each feature on prediction accuracy by randomly shuffling individual features and measuring the resulting performance drop. The refined PMI findings (Fig. 10) were consistent with those from the initial multi-model analysis (Fig. 6C), reinforcing the reliability of top-ranked features such as ClinPred, am_pathogenicity, and DEOGEN2_rankscore. It also provided further evidence of the model’s reliability and interpretability. All interpretability steps were implemented in Python using the lime and sklearn.inspection.permutation_importance packages.

### External testing

Finally, and to evaluate generalizability, we assembled an independent validation dataset from the ClinGen Variant Curation Expert Panel resource (https://clinicalgenome.org/). This set contained curated missense variants that were not included in training. Predictions were generated using our best-performing Extra Trees model and benchmarked against widely used pathogenicity predictors, including MetaRNN, REVEL, ClinPred, and others as shown in Supplementary Table S8. Model outputs were harmonized into binary pathogenic versus benign calls for direct comparison, and performance was assessed using accuracy, precision, recall, specificity, F1 score, MCC, and confusion matrix counts. The performance of our model compared to other tools is summarized in Table 7.

**Table 7:** Performance comparison of the extra trees classifier against established pathogenicity predictors on an independent dataset

**Predictor**	**Accuracy**	**Precision**	**Recall**	**Specificity**	**F1**	**MCC**	**TP**	**TN**	**FP**	**FN**	**Missed**
**Our Extra Trees Model**	**0.99122807**	**0.996825397**	**0.990536278**	**0.992805755**	**0.993670886**	**0.979437666**	**314**	**138**	**1**	**3**	**0**
**MetaRNN**	0.788546	0.91634981	0.765079	0.841727	0.833910035	0.566523558	241	117	22	74	2
**REVEL**	0.751101	0.907258	0.714285714	0.834532374	0.79928952	0.508079466	225	116	23	90	2
**ClinPred**	0.755506608	0.935897436	0.695238095	0.892086331	0.797814208	0.541653397	219	124	15	96	2
**PolyPhen**	0.726872247	0.835087719	0.755555556	0.661870504	0.793333333	0.397993934	238	92	47	77	2
**SIFT**	0.72406181	0.832167832	0.755555556	0.652173913	0.792013311	0.388978564	238	90	48	77	3
**MutationAssessor**	0.711217	0.803636364	0.767361111	0.58778626	0.785079929	0.346651355	221	77	54	67	37
**MetaLR**	0.740088106	0.937777778	0.66984127	0.899280576	0.781481481	0.52463702	211	125	14	104	2
**MetaSVM**	0.726872247	0.956937799	0.634920635	0.935252	0.763358779	0.527245289	200	130	9	115	2
**PROVEAN**	0.56440281	0.793296089	0.487972509	0.727941176	0.604255319	0.203865512	142	99	37	149	29

Metrics include accuracy, precision, recall, specificity, F1 score, MCC, and confusion matrix counts (TP, TN, FP, FN). The Extra Trees model achieved superior accuracy and MCC, with markedly fewer misclassifications than existing tools.

*Note:* Bold values are the highest values representing the top performing metrics.

## Results

The results of our study are detailed below, encompassing model performance evaluation, statistical validation, feature importance, interpretability analysis, and threshold optimization. Each aspect of the analysis is explained to highlight its role in evaluating predictor performance and relevance to genetic variant classification.

### Feature selection and engineering

**Figure 2:**
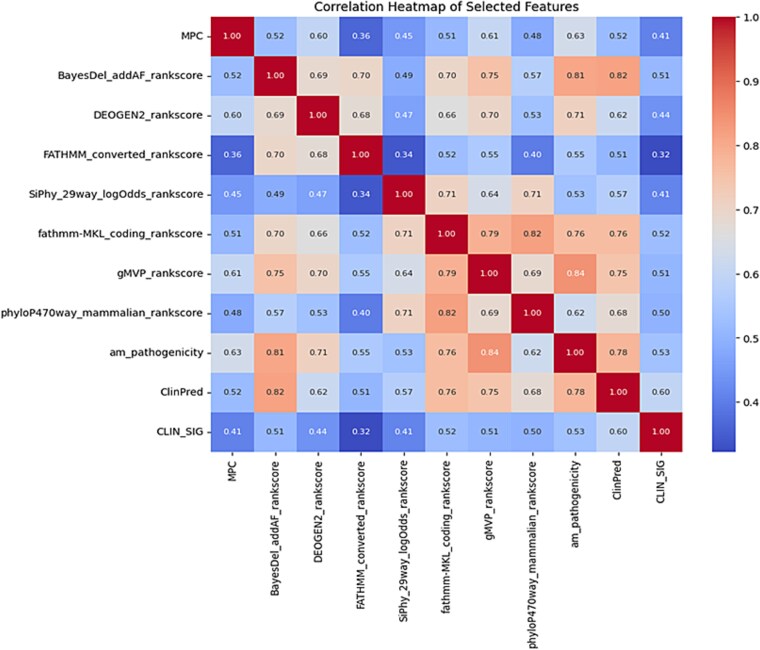
The heatmap correlation of the top features.

The correlation heatmap shown in Fig. 2, illustrates the relationships among various predictive scores used in pathogenicity classification. Certain features, such as BayesDel and ClinPred, exhibit strong correlations, indicating potential redundancy, while others, like MPC and phyloP, show weaker associations. Moderate correlations with clinical significance suggest that multiple factors contribute to pathogenicity classification rather than a single dominant predictor. The presence of collinearity among some features highlights the importance of careful feature selection to optimize model performance and interpretability. These findings reinforce the need for a multi-feature approach in predictive modeling.

**Figure 3:**
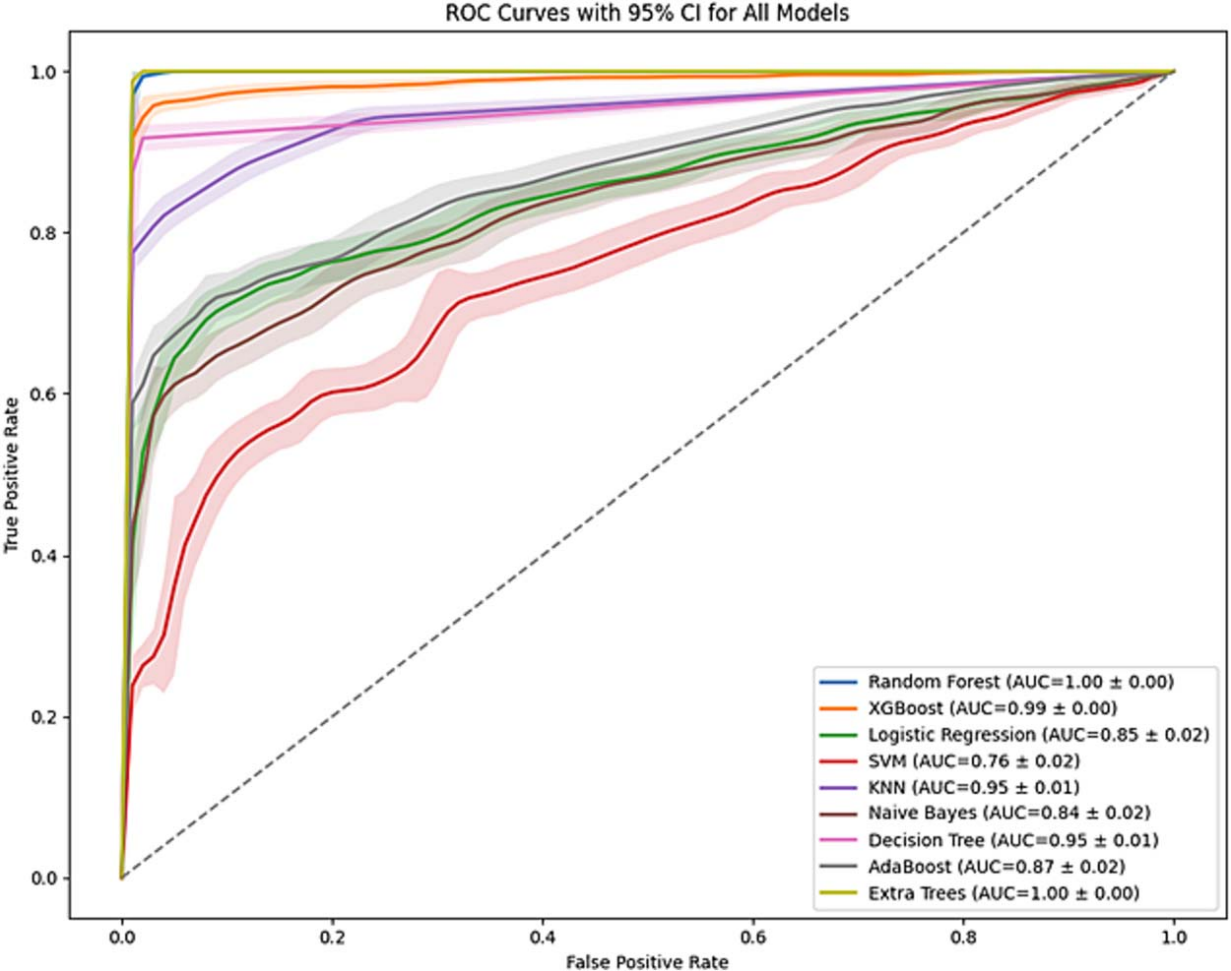
ROC curves with 95% confidence intervals for all models, highlighting the performance variability across models.

**Figure 4:**
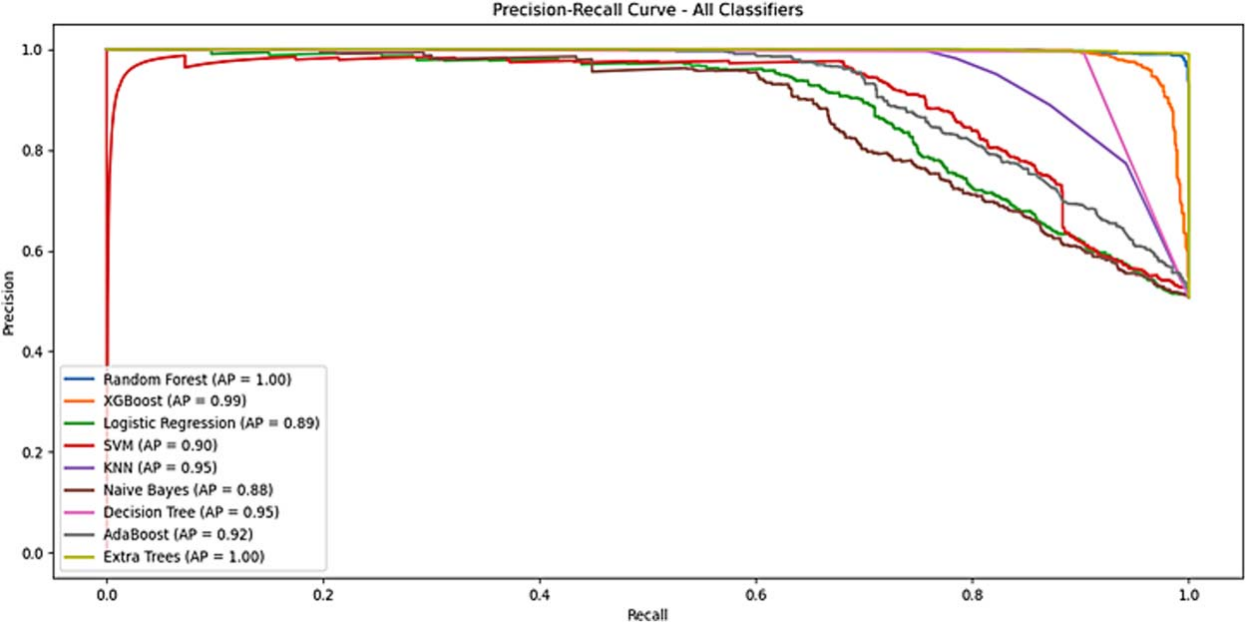
The precision-recall curve of the models.

### Model development and evaluation/threshold optimization and prediction

The performance of nine models-Random Forest, XGBoost, Logistic Regression, SVM, KNN, Naive Bayes, Decision Tree, AdaBoost, and Extra Trees-was evaluated based on several metrics, including AUC, precision, recall, F1 score, sensitivity, specificity, MCC, and Cohen’s kappa. These metrics were selected to comprehensively assess the models’ ability to distinguish between pathogenic and benign genetic variants. ROC curves with 95% confidence intervals are shown in Fig. 3. Extra Trees, Random Forest, and XGBoost achieved near-perfect AUCs (1.00, 1.00, and 0.99, respectively), demonstrating consistent performance with minimal variability across folds. Models like SVM and Naive Bayes showed wider confidence bands, indicating performance instability. The inclusion of confidence intervals enhances model reliability assessment and supports robust classifier selection. While the precision-Recall Curves illustrated the models’ ability to identify pathogenic variants though minimizing false positives. The ROC-AUC and Precision-Recall plots are shown in Figs. 3 and 4 below. The AUC, precision, and recall were prioritized in calculating the weighted scores due to their relevance in medical applications, where accurate detection of pathogenic variants and minimizing false positives are essential. Among the models, Extra Trees achieved the highest weighted score (0.9803), followed closely by Random Forest (0.9653) and XGBoost (0.9597). Logistic Regression, while effective in general, had a lower weighted score (0.7852), reflecting its reduced adaptability to this dataset. Similarly, Naive Bayes, known for its simplicity, performed sub-optimally (0.7695). These results underscore the superiority of ensemble-based methods, particularly Extra Trees, for handling the complex relationships inherent in genetic data. Table 1 summarizing the weighted scores is shown below.

**Table 1:** The weighted score of each model

**Predictor**	**Weighted score**
Random Forest	0.9653
XGBoost	0.9597
Logistic Regression	0.7852
SVM	0.8399
KNN	0.9014
Naïve Bayes	0.7695
Decision Trees	0.9489
AdaBoost	0.8101
Extra Trees	**0.9803**

*Note:* Bold values are the highest values representing the top performing metrics.

### Multi-seed evaluation and best seed selection

To minimize performance variability and ensure consistency in evaluation, each classifier was trained and validated across multiple random seeds. Performance metrics such as AUC, accuracy, and F1-score were computed for each run, and the seed yielding the highest AUC was designated as optimal for that model. As shown in Table 2, ensemble models such as Extra Trees and Random Forest exhibited stable performance across seeds, with minimal deviation in AUC. By contrast, some traditional models like SVM and Logistic Regression showed moderate variability.

**Table 2:** Best-performing random seed and corresponding AUC for each model based on multi-seed evaluation

**Model**	**Best seed**	**Best AUC**	**Random seeds**				
			**Seed_42**	**Seed_101**	**Seed_202**	**Seed_303**	**Seed_404**
**Random Forest**	42	1.00	1.00	0.999	0.998	0.997	0.999
**XGBoost**	42	0.99	0.99	0.985	0.981	0.98	0.985
**Logistic Regression**	101	0.85	0.82	0.85	0.83	0.81	0.82
**SVM**	101	0.76	0.74	0.76	0.75	0.72	0.73
**KNN**	202	0.95	0.92	0.94	0.95	0.93	0.94
**Naive Bayes**	101	0.84	0.83	0.84	0.82	0.81	0.83
**Decision Tree**	202	0.95	0.93	0.94	0.95	0.93	0.94
**AdaBoost**	101	0.87	0.85	0.87	0.86	0.85	0.86
**Extra Trees**	42	1	1	0.999	0.999	0.998	0.999

Using these optimal seeds, the models were re-evaluated for final performance reporting, confidence interval estimation, and interpretability analysis. This two-phase evaluation ensures that the final conclusions are not biased by initialization effects and represent the best configuration for each algorithm.

The evaluation of the models revealed key insights into the performance which included several metrics. Almost all models demonstrated high predictive performance, with some differences among them. Table 3 summarize the performance metrics for each model, including accuracy, precision, recall, F1-score, Cohen’s kappa, and MCC.

**Table 3:** The performance metrics of the models after splitting 80:20

	**Random forest**	**XGBoost**	**Logistic regression**	**SVM**	**KNN**	**Naive bayes**	**Decision tree**	**AdaBoost**	**Extra trees**
**Accuracy**	0.998877	0.990168	0.850484	0.87966	0.943237	0.836732	0.94931	0.845901	**0.999235**
**Precision**	0.996028	0.994012	0.782931	0.935142	0.950837	0.765683	0.996008	0.902582	**0.993396**
**Recall**	0.906872	0.900542	0.76311	0.716998	0.821881	0.750452	0.902351	0.695298	**0.95208**
**F1-score**	0.949361	0.944972	0.772894	0.811668	0.881668	0.757991	0.946869	0.785495	**0.972299**
**Specificity**	0.996269	0.994403	0.781716	0.948694	0.956157	0.76306	0.996269	0.922575	**0.99347**
**Sensitivity**	0.906872	0.900542	0.76311	0.716998	0.821881	0.750452	0.902351	0.695298	**0.95208**
**Cohens kappa**	0.90186	0.893613	0.544608	0.663239	0.776368	0.51335	0.897281	0.615631	**0.944926**
**MCC**	0.905601	0.897706	0.544788	0.682535	0.78381	0.513455	0.901391	0.632969	**0.94577**
**FPR**	0.003731	0.005597	0.218284	0.051306	0.043843	0.23694	0.003731	0.077425	**0.00653**
**TNR**	0.996269	0.994403	0.781716	0.948694	0.956157	0.76306	0.996269	0.922575	**0.99347**
**TPR**	0.906872	0.900542	0.76311	0.716998	0.821881	0.750452	0.902351	0.695298	**0.95208**

*Note:* Bold values are the highest values representing the top performing metrics.

To ensure the robustness of the findings in Tables 4 and 5-fold cross-validation was applied. This method splits the dataset into training and validation sets multiple times, ensuring that every data point is used for both training and testing. Cross-validation helps mitigate overfitting and provides a more reliable estimate of a model’s generalizability. The results of both methods showed consistent performance for the top models (Extra Trees, Random Forest, and XGBoost) with accuracy of approximately 0.99 as shown earlier in Tables 3 and 4, confirming their stability and reliability. In addition to cross-validation, 95% confidence intervals were estimated for each performance metric using bootstrapping (*n* = 1000 iterations). This provides a more robust assessment of metric variability and strengthens confidence in model reliability. The results are summarized in Table 5, where ensemble models-particularly Extra Trees-consistently demonstrated narrow confidence intervals, reflecting stable and reproducible performance across resampled subsets.

**Table 4:** The performance metric results after the 5-fold cross validation

	**Random forest**	**XGBoost**	**Logistic regression**	**SVM**	**KNN**	**Naive bayes**	**Decision tree**	**AdaBoost**	**Extra trees**
**Accuracy**	0.998098	0.989473	0.848256	0.887864	0.94448	0.8391	0.951576	0.847849	**0.998543**
**Precision**	0.990017	0.99127	0.774275	0.936298	0.945716	0.760178	0.991372	0.895921	**0.987785**
**Recall**	0.914481	0.900886	0.767874	0.720471	0.82222	0.75777	0.911006	0.704598	**0.953597**
**F1-score**	0.950728	0.943893	0.771009	0.814308	0.879626	0.758938	0.949464	0.788761	**0.970348**
**Specificity**	0.990846	0.992131	0.776208	0.9509	0.95275	0.760874	0.992146	0.918017	**0.988288**
**Sensitivity**	0.914481	0.900886	0.767874	0.720471	0.82222	0.75777	0.911006	0.704598	**0.953597**
**MCC**	0.908009	0.896787	0.544023	0.68991	0.781633	0.518589	0.906154	0.637329	**0.942545**
**Cohens kappa**	0.905332	0.893017	0.543932	0.671292	0.774892	0.51854	0.903129	0.622553	**0.941908**

*Note:* Bold values are the highest values representing the top performing metrics.

**Table 5:** Performance metrics with 95% confidence intervals estimated via bootstrapping

	**AUC (CI)**	**Precision (CI)**	**Recall (CI)**	**F1 (CI)**	**Kappa (CI)**	**MCC (CI)**
**Random Forest**	**0.999 [0.998–0.999]**	0.993 [0.987–0.998]	0.912 [0.895–0.930]	0.951 [0.942–0.960]	0.905 [0.887–0.922]	0.908 [0.891–0.924]
**XGBoost**	0.988 [0.984–0.992]	**0.996 [0.992–0.999]**	0.893 [0.875–0.912]	0.942 [0.931–0.952]	0.888 [0.869–0.907]	0.893 [0.875–0.910]
**Logistic Regression**	0.850 [0.834–0.866]	0.788 [0.763–0.813]	0.765 [0.738–0.789]	0.776 [0.757–0.796]	0.552 [0.517–0.587]	0.552 [0.518–0.587]
**SVM**	0.756 [0.736–0.777]	0.699 [0.670–0.727]	0.637 [0.610–0.665]	0.666 [0.643–0.689]	0.353 [0.313–0.392]	0.355 [0.313–0.394]
**KNN**	0.948 [0.940–0.957]	0.956 [0.942–0.968]	0.821 [0.799–0.844]	0.884 [0.868–0.898]	0.781 [0.754–0.808]	0.789 [0.763–0.814]
**Naive Bayes**	0.835 [0.819–0.852]	0.766 [0.742–0.791]	0.752 [0.724–0.776]	0.759 [0.738–0.779]	0.514 [0.479–0.550]	0.514 [0.479–0.550]
**Decision Tree**	0.954 [0.945–0.962]	0.991 [0.985–0.997]	0.916 [0.898–0.932]	0.952 [0.942–0.961]	0.906 [0.889–0.923]	0.909 [0.892–0.925]
**AdaBoost**	0.874 [0.860–0.889]	0.904 [0.884–0.924]	0.702 [0.675–0.730]	0.790 [0.771–0.810]	0.623 [0.593–0.655]	0.640 [0.611–0.670]
**Extra Trees**	**0.999 [0.998–1.000]**	0.992 [0.986–0.997]	**0.958 [0.946–0.969]**	**0.975 [0.968–0.981]**	**0.949 [0.937–0.963]**	**0.950 [0.938–0.963]**

*Note:* Bold values are the highest values representing the top performing metrics.

### Statistical validation

**Figure 5:**
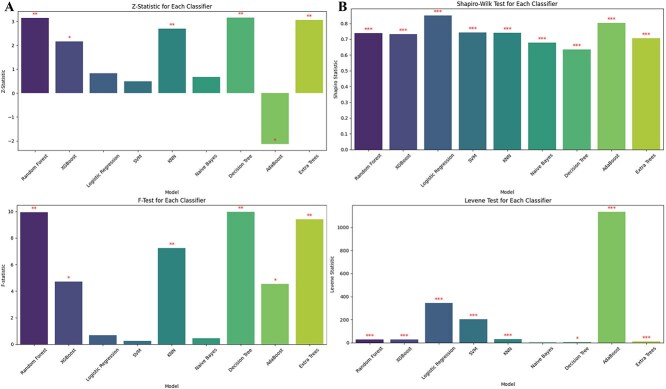
(A) The *Z*-statistics and *F*-test statistical analysis plots of all models. (B) The Shapiro–Wilk and Levene statistical analysis plot of all models.

Several statistical tests were performed to validate differences in models’ performance and ensure the reliability of the results. The *z*-test was used to compare the mean performance metrics of the models. Random Forest, Decision Tree, and Extra Trees exhibited significant *z*-statistics (*P* < .01), indicating that their performance metrics were significantly higher than others. Shapiro–Wilk Test assessed the normality of the predicted probabilities for each predictor. While most models showed deviations from normality (*P* < .05), the test highlighted the distributional differences, supporting the need for additional robust metrics. Levene’s Test evaluated the homogeneity of variances between models. It revealed that Extra Trees and Random Forest had more stable variances across folds compared to Logistic Regression and Naive Bayes, further supporting their robustness. The *F*-test was conducted to compare the means of performance metrics across models. Random Forest, Decision Tree, and Extra Trees showed statistically significant differences (*P* < .01), confirming their superior performance. These statistical tests were essential in validating that the observed differences in predictor performance were not due to random variation but were statistically meaningful. The plots of all four test is shown in Fig. 5A and B below.

**Figure 6:**
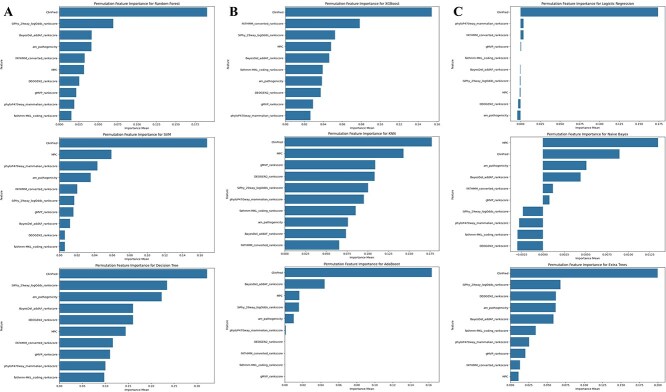
(A) The PMI plots for random Forest, SVM, and decision tree. (B) The PMI plots for XGBoost, KNN, and AdaBoost. (C) The PMI plots for logistic regression, naïve Bayes and extra trees.

**Figure 7:**
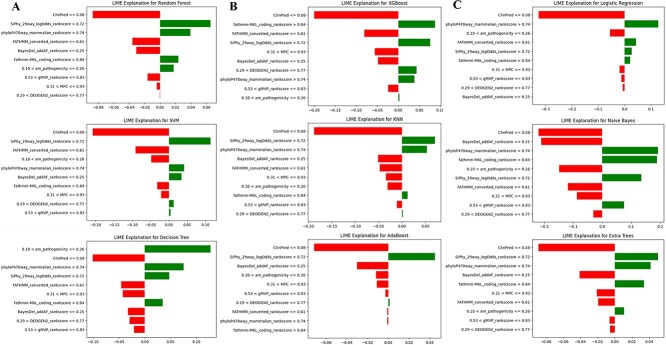
(A) The LIME plots for random Forest, SVM, and decision tree. (B) The LIME plots for XGBoost, KNN, and AdaBoost. (C) The LIME plots for logistic regression, naïve Bayes, and Extra Trees.

**Figure 8:**
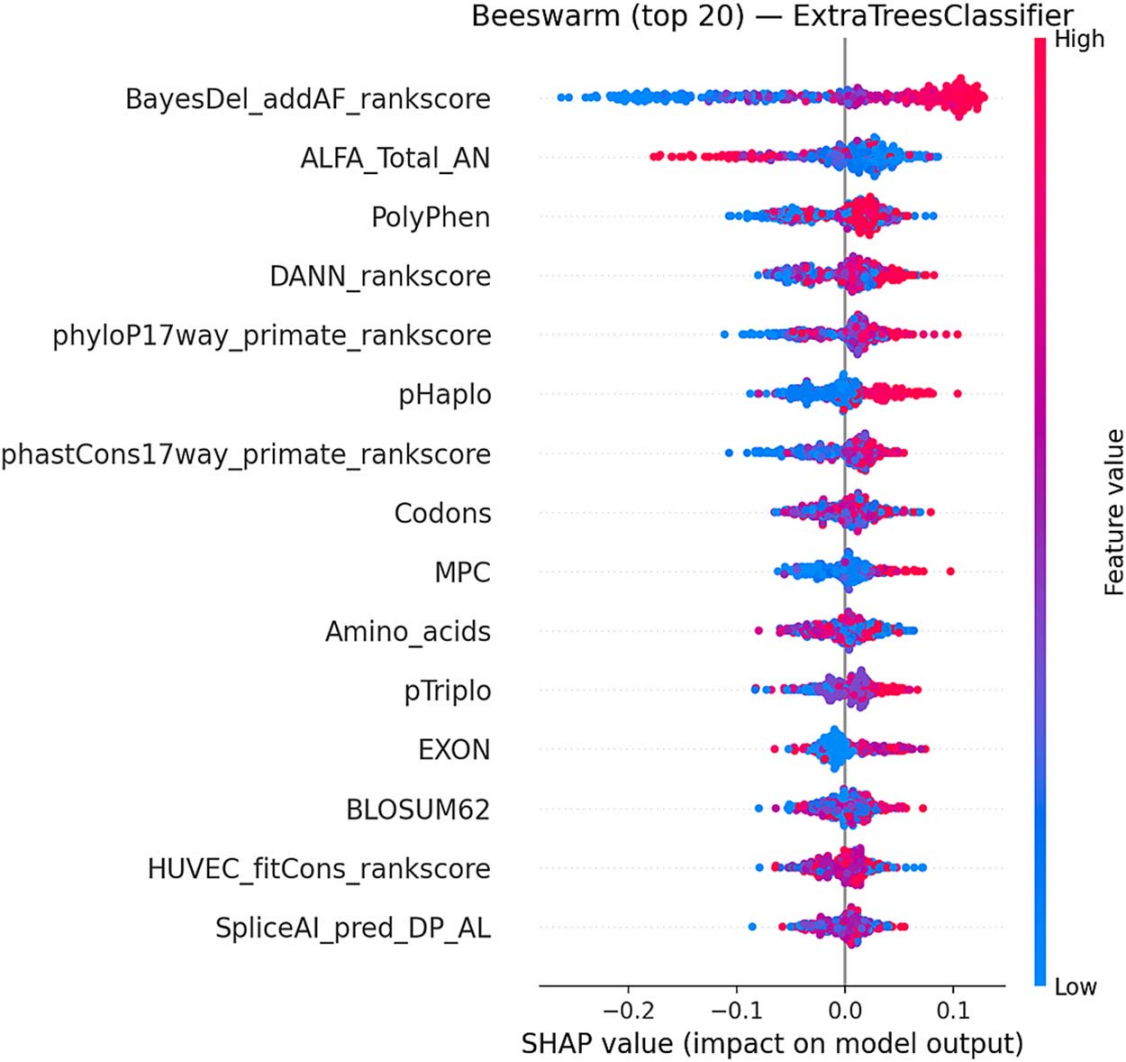
Global SHAP beeswarm plot (top 20 features) for the extra trees classifier. Each point represents a variant, with colors denoting feature values (blue = low, red = high). Features such as BayesDel, allele frequency metrics, conservation scores, and protein-context predictors were the strongest contributors to model output.

**Figure 9:**
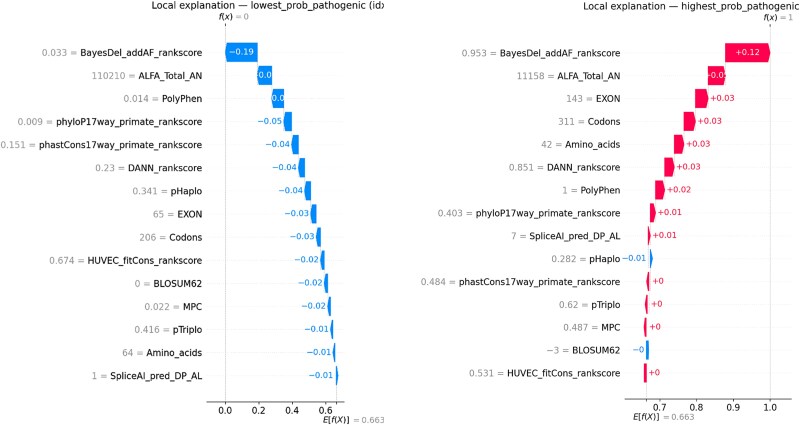
Local SHAP waterfall plots for representative variants. (Left side) lowest-probability pathogenic example showing negative contributions from meta-predictors and conservation scores, consistent with a benign classification. (Right side) highest-probability pathogenic example where strong positive contributions from BayesDel, PolyPhen, and conservation metrics outweighed context features, driving a pathogenic classification.

### Model interpretability and reliability assessment

Feature importance was analyzed using RFE and PMI. The analysis revealed that ClinPred, SiPhy_29way_logOdds_rankscore, fathmm-MKL_coding_rankscore, phyloP470way_mammalian_rankscore, and MPC as the top features. These features were consistently identified across models therefore are considered critical determinants for classifying variants. The permutation feature importance plots for each model are provided in Fig. 6A, B, and C. To provide interpretability, we applied LIME to visualize the impact of these features on individual predictions. For instance, ClinPred consistently had the highest contribution to predictions across models. Similarly, SiPhy_29way_logOdds_rankscore and phyloP470way_mammalian_rankscore were crucial for identifying pathogenic variants. Permutation importance analysis corroborated these findings by quantifying the impact of each feature on model performance. The LIME plots of each model are provided in Fig. 7A, B, and C.

Global SHAP analyses of the best model (Extra Trees) showed that meta-predictor scores (e.g. BayesDel), evolutionary constraint (phyloP, phastCons), and protein-context features (MPC, BLOSUM62, Codon/Exon annotations) were the dominant drivers of predictions as shown in Fig. 8. Higher values of these meta-predictors and constraint metrics pushed predictions toward pathogenicity, whereas low constraint and benign-leaning amino acid context pushed predictions toward benign. The global SHAP ranking was consistent with permutation importance and LIME, supporting a coherent feature importance profile across methods.

Local SHAP waterfall plots provided case-level explanations as shown in Fig. 9. In a high-confidence pathogenic example, large positive contributions from BayesDel, DANN, and PolyPhen outweighed smaller negative contributions from context features, yielding a high predicted probability. Conversely, a low-probability benign example showed uniformly negative contributions from the same feature families, explaining why the prediction fell below the decision threshold. Misclassified or borderline variants typically showed conflicting evidence-such as a high meta-predictor score but weak conservation-clarifying failure modes and suggesting where additional functional evidence would be most informative.

Across methods, ClinPred (and related ensemble meta-scores) consistently ranked highest, with constraint scores (phyloP/phastCons) and protein-context features (MPC) providing complementary signal. Misclassified cases in LIME/SHAP typically showed borderline or conflicting evidence across these feature families, suggesting where additional functional evidence or re-weighting may help.

### Selection of the best predictor

Decision thresholds for each classifier were optimized using ROC and PR analyses. The final cutoffs were selected evaluated using Youden’s J and F1-optimal criteria with Youden’s J applied as a tiebreaker, and stability confirmed through 5-fold cross-validation. Table 6 reports the optimal thresholds used for classification, while Supplementary Table S6 provides the full benchmarking results, including AUC values and alternative threshold criteria (Youden’s J and F1-optimal). Across models, ensemble tree methods (Extra Trees, Random Forest, XGBoost) consistently outperformed linear and kernel-based approaches, with Extra Trees achieving the highest performance (AUC = 0.9997, threshold = 0.20).

**Table 6:** The thresholds for each model

**Predictor**	**Threshold**
**Random Forest**	0.28
**XGBoost**	0.25
**Logistic Regression**	0.68
**SVM**	0.37
**KNN**	0.60
**Naïve Bayes**	1.00
**Decision Trees**	1.00
**AdaBoost**	0.49
**Extra Trees**	0.20

**Figure 10:**
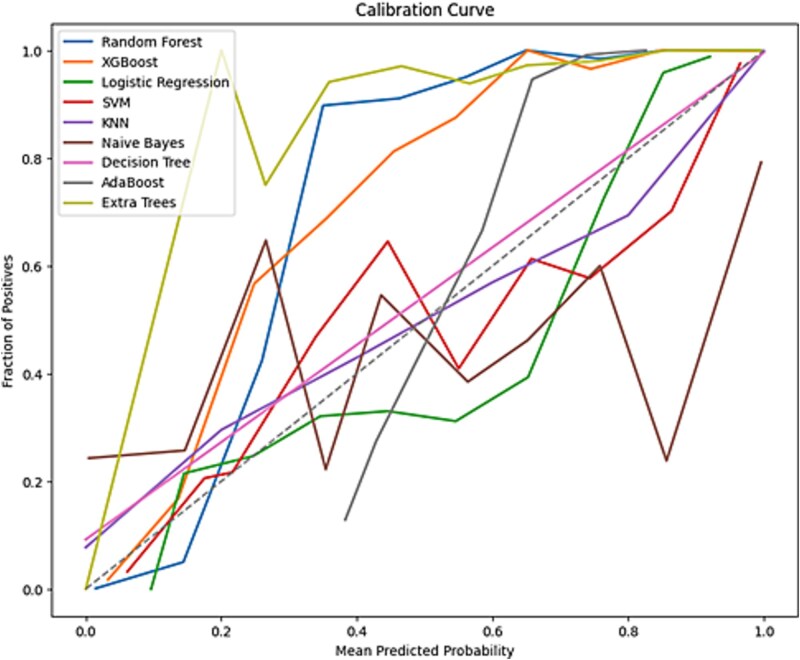
The calibration curve of all models.

A Calibration curve was generated to evaluate the reliability of the predicted probabilities for each predictor. Extra Trees and Random Forest showed strong calibration, with predicted probabilities aligning closely with observed outcomes, as shown in Fig. 10. This alignment is critical in clinical settings, as poorly calibrated models may lead to overconfidence in incorrect predictions, increasing the risk of misclassification. Well-calibrated models ensure that a variant classified with 90% pathogenicity probability truly corresponds to pathogenic cases in 90% of real-world scenarios, preventing unnecessary medical interventions or missed diagnoses. Calibration is particularly important for integrating ML predictions into clinical workflows, such as genetic counseling, ACMG-AMP classification, and risk assessment, where probability estimates influence decisions on surveillance, confirmatory testing, or risk-reduction strategies [27]. Calibration analysis revealed notable differences in the reliability of probability estimates across models as shown in Fig. 10. The tree-ensemble classifiers, particularly Extra Trees and Random Forest, showed the strongest calibration, with predicted probabilities closely aligned to observed frequencies across most ranges. Both exhibited slight over-confidence at the extremes, where predicted probabilities exceeded observed outcomes. Logistic Regression tended to be under-confident in the mid-range, whereas SVM, KNN, Decision Tree, and AdaBoost produced unstable, jagged curves that reflected poor calibration and unreliable probability estimates. These patterns suggest that ensemble methods not only deliver the highest accuracy but also provide the most trustworthy probability estimates for clinical interpretation, minimizing the risk of over- or under-calling variant pathogenicity.

**Figure 11:**
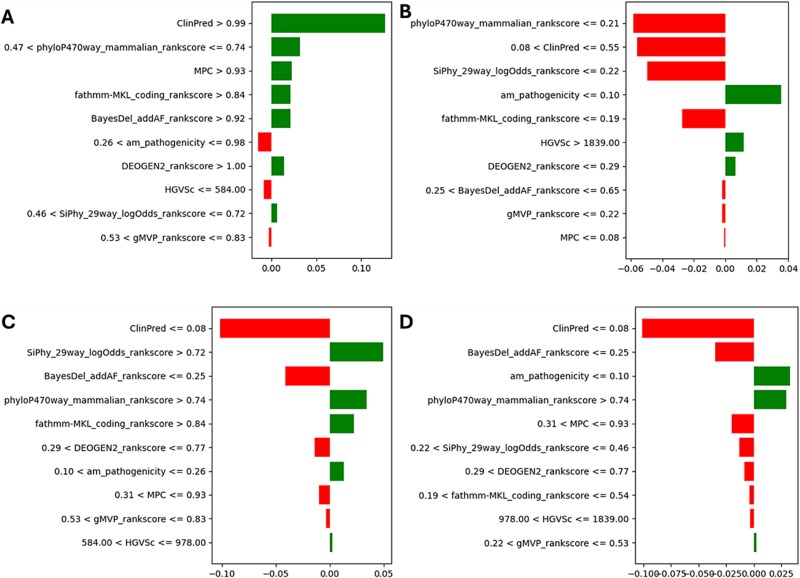
LIME explanations for representative predictions by the extra trees model. (A) TP, (B) TN, (C) FP, (D) FN.

**Figure 12:**
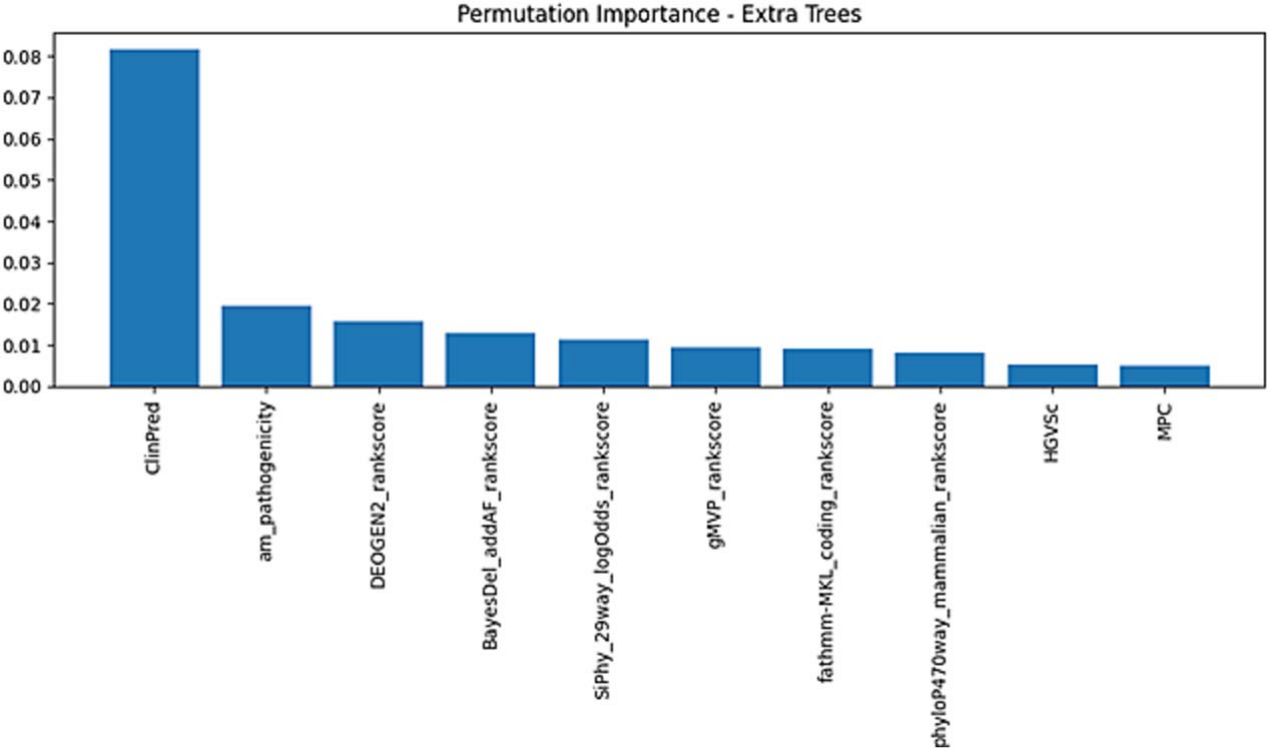
Refined permutation importance plot for extra trees using the best-performing seed.

To build upon our previous interpretability analysis, we performed a more focused evaluation of the Extra Trees model using the best-performing seed identified during multi-seed analysis. LIME was applied to four representative prediction outcomes: a TP, TN, FP, and FN. These cases provide a detailed view of how individual features contributed to the model’s decisions across both correct and incorrect classifications. As shown in Fig. 11, features like ClinPred, phyloP470way_mammalian_rankscore, and MPC consistently influenced the prediction outcomes. Notably, in misclassified samples (Fig. 11C and D), deviations in these key features appeared to drive incorrect predictions, highlighting areas for potential refinement in model training or dataset balancing.

To strengthen the interpretability of the best-performing model, we recalculated the PMI for the Extra Trees classifier using the best-performing seed identified through multi-seed evaluation. As illustrated in Fig. 12, ClinPred consistently emerged as the most influential feature, followed by am_pathogenicity, DEOGEN2_rankscore, and BayesDel_addAF_rankscore. These results reinforce our prior findings and confirm that these features play central roles in driving pathogenicity predictions.

In contrast to the earlier PMI results performed across three models (Logistic Regression, Naive Bayes, and Extra Trees), which are displayed in Fig. 6C, this refined analysis (Fig. 12) uses a consistent seed and model configuration to ensure stability and reproducibility. While ClinPred remained the top-ranked feature across both analyses, this update reduces inter-model variability and offers clearer insight into the specific contribution of each feature under optimal conditions. The alignment between prior and current PMI rankings further validates the robustness of our feature selection pipeline and highlights the biological significance of top-ranked features like ClinPred, SiPhy_29way_logOdds, and phyloP470way_mammalian in breast cancer variant classification. ClinPred’s prominence likely reflects its meta-predictor design aggregating conservation and functional evidence; its high correlation with other ensemble scores (e.g. BayesDel) indicates overlapping information. Constraint measures (phyloP/phastCons) contribute to an orthogonal signal, explaining their recurrent secondary importance. Naive Bayes down-weights ClinPred relative to other models, consistent with its feature-independence assumption and the observed collinearity.

### External testing

On the independent testing dataset, our Extra Trees model substantially outperformed existing predictors across all evaluation metrics as shown in Table 7. Extra Trees achieved 99.1% accuracy, 0.994 F1 score, and an MCC of 0.98, with only four misclassified variants out of 456. In comparison, MetaRNN, REVEL, and ClinPred showed accuracies in the range of 75%–79%, with markedly lower recall and MCC values. While these established tools misclassified between 74–96 pathogenic variants, Extra Trees achieved near-complete sensitivity with only three false negatives and one false positive. Supplementary Table S8 illustrates representative variant-level predictions, confirming the consistency of Extra Trees classifications with clinical annotations. These results demonstrate the superior robustness and clinical readiness of our model relative to state-of-the-art meta-predictors.

## Discussion

This study evaluated multiple ML models for predicting the pathogenicity of missense variants using a breast cancer disease-gene-specific dataset. The results highlight the robustness of ensemble-based methods, particularly the Extra Trees classifier, which consistently outperformed others in handling the complexity of high-dimensional genomic data. Compared to traditional models such as Logistic Regression and Naive Bayes, ensemble techniques better captured non-linear interactions between genomic features. These findings are consistent with previous research showing the effectiveness of ensemble methods in genomic classification tasks [28]. Among the ensemble methods, Extra Trees surpassed both Random Forest and XGBoost in performance. This advantage is attributed to its randomized threshold selection strategy during tree construction, which reduces variance and enhances generalization [29]. Such behavior is particularly useful in pathogenicity prediction, where genomic features often exhibit interdependencies and redundancy. While XGBoost remains a powerful classifier, it requires extensive hyperparameter tuning and is computationally intensive [30]. In contrast, Extra Trees offers a favorable balance between performance and efficiency, making it more suitable for practical clinical deployment. The feature selection process-integrating Pearson correlation filtering with RFE, proved critical in enhancing predictive performance. Features such as ClinPred, fathmm-MKL_coding_rankscore, and phyloP470way_mammalian_rankscore consistently emerged as top models, reinforcing their known biological relevance [31]. Interpretability was prioritized using LIME and PMI, which helped clarify the decision logic of the model. This is crucial in a clinical setting, where explainable AI is needed to gain trust from healthcare professionals and meet regulatory standards. Interpretability analyses further reinforced these findings. In Fig. 6, ClinPred exhibited the highest importance ratio across all classifiers except Naïve Bayes, reflecting its role as an ensemble meta-predictor that integrates multiple evidence streams. This dominance was replicated in the refined PMI analysis of Extra Trees (Fig. 12), where ClinPred, BayesDel, and constraint scores (phyloP, phastCons) consistently ranked among the strongest predictors. Importantly, LIME explanations complemented these global importance results by providing case-level insights, showing that high ClinPred or BayesDel values systematically pushed predictions toward pathogenicity, while weak conservation or benign amino acid contexts pulled them toward benign. Together, PMI and LIME establish a coherent interpretability profile, confirming both global feature drivers and their variant-level effects. These aligned findings not only validate the biological relevance of top features but also support clinical trust in model decisions, an essential step for translational deployment. To ensure statistical rigor, the performance of Extra Trees was validated using cross-validation and hypothesis testing. Although we did not include explicit test statistics in the discussion text, *z*-tests, ANOVA, and Levene’s test were employed in the methods to assess significance and model stability. Extra Trees maintained superior performance across multiple dataset splits, confirming its generalizability. Furthermore, calibration curves confirmed reliability through by producing well-calibrated probability estimates-an essential factor for clinical decision-making. Importantly, these results build upon our prior work, which benchmarked existing genome-wide tools and achieved a maximum accuracy of 0.73 on the same dataset’s structure and sources [22]. This underscores the value of disease-specific modeling and highlights the need for tailored tools that can surpass generic models in specialized clinical contexts. Importantly, validation on an independent dataset confirmed that Extra Trees not only excelled on internal cross-validation but also generalized to external data. Its performance surpassed widely adopted meta-predictors, including MetaRNN, REVEL, and ClinPred, underscoring the value of disease-specific modeling. This external benchmarking strengthens confidence in its translational potential for breast cancer variant interpretation. In conclusion, Extra Trees not only achieves high accuracy but also provides reliable and interpretable predictions, making it an ideal candidate for variant classification in breast cancer. Its ability to handle feature dependencies, minimize overfitting, and maintain confidence calibration positions it as a strong foundation for future development. Looking ahead, hybrid models that incorporate deep learning while preserving interpretability-such as attention mechanisms or shallow-deep ensembles-may offer a promising direction for further improvement. The demonstrated results suggest that the Extra Trees model could be integrated into clinical pipelines for variant interpretation in hereditary breast cancer, following further validation. Future work will involve external validation on breast cancer cohorts and might be extended to the integration with electronic health records for decision support.

## Conclusion

This study demonstrates that ensemble-based ML models-particularly the Extra Trees classifier-offer a robust and interpretable framework for predicting the pathogenicity of breast cancer missense variants when trained on a curated disease-gene-specific dataset. The Extra Trees model consistently outperformed conventional models such as Logistic Regression and Naive Bayes across all performance metrics, demonstrating superior calibration, and generalizability. Its randomized feature splitting mechanism effectively reduced overfitting, while calibration curves validated the reliability of its probability outputs-both essential for clinical adoption. A major strength of this work lies in its emphasis on interpretability. By incorporating LIME and PMI, the study provided transparent insights into model decision-making and highlighted biologically meaningful features such as ClinPred, fathmm-MKL coding rank score, and phyloP470way mammalian rank score. These insights strengthen the translational relevance of the model and align predictive performance with biological plausibility. Moreover, the study underscores the value of disease-specific datasets in precision medicine. Models tailored to the genetic architecture of specific diseases-such as breast cancer-achieved higher classification accuracy and reduced both false positives and false negatives, enhancing their utility for clinical diagnostics. Looking forward, future work should expand dataset diversity by including variants from underrepresented populations to improve model generalizability. Additionally, integrating deep learning techniques for automated feature extraction with interpretable ensemble models could yield even more powerful hybrid frameworks. In conclusion, the Extra Trees model emerged as the most clinically promising tool for pathogenicity prediction-combining accuracy, interpretability, and computational efficiency. External validation against an independent testing dataset demonstrated superior performance of Extra Trees over established tools, highlighting its readiness for integration into clinical variant interpretation pipelines. These findings contribute to the growing field of AI-driven precision medicine and lay the groundwork for clinically deployable tools in genetic diagnostics. To conclude, our model was specifically developed for a curated panel of 107 breast cancer–associated genes, ensuring disease-relevant training, and clinical applicability. While the current scope is limited to breast cancer missense variants, the underlying ML framework is adaptable. With appropriate disease-specific variant datasets, the pipeline can be extended to other hereditary cancers or complex diseases, enabling tailored pathogenicity prediction beyond breast cancer. This highlights both the focused strength of the present study and its potential for broader generalization in precision oncology.

**Key Points**

- Disease-specific ML models outperform genome-wide tools in predicting the pathogenicity of breast cancer missense variants, offering greater precision, and clinical relevance.
- The Extra Trees classifier achieved the highest performance (AUC = 0.999, CI [0.998–1.000]) using a curated breast cancer-specific dataset with conservation, frequency, and functional features.
- Model transparency was ensured through interpretability tools like LIME and permutation importance, supporting clinical trust and understanding of prediction rationale.
- Robust validation through cross-validation, multi-seed testing, and calibration curves confirmed the model’s stability, reliability, and readiness for clinical deployment.

## Supplementary Material

Supplementary_materials_bbaf611(1)

## Data Availability

Datasets prepared and used in this research were downloaded from online databases including COSMIC (https://cancer.sanger.ac.uk/cosmic), CBioPortal (https://www.cbioportal.org/datasets), BRCAExchange (https://brcaexchange.org/), TCGA (https://www.cancer.gov/ccg/research/genome-sequencing/tcga), ClinVar (https://www.ncbi.nlm.nih.gov/clinvar/), and HGMD Professional 2024.3 (https://www.hgmd.cf.ac.uk/). The final data structure used in this research is available on the GitHub repository. The final dataset is not publicly available due to the inclusion of data from the HGMD Professional version 2024.3 which is subscription based. An annotated, ready to test dataset was collected from the Clingen dataset (https://clinicalgenome.org/) and is provided with the GitHub Repository for testing the workflow. The code used to generate the results is available on https://github.com/rahafahmad89/Precision-for-prediction.
