# Supplementary material for: Precision in prediction: tailoring machine learning models for breast cancer missense variants pathogenicity prediction
Source: Brief Bioinform. 2025 Nov 20;26(6):bbaf611. doi: 10.1093/bib/bbaf611 (PMC12632190; doi:10.1093/bib/bbaf611)
Supplement: Supplementary_materials_bbaf611(1) [file supplementary_materials_bbaf611(1).zip › Supplementary_materials_bbaf611/Supplementary.docx]

**Supplementary Information for Precision in Prediction: Tailoring Machine Learning Models for Breast Cancer Missense Variants Pathogenicity Prediction**

Rahaf M. Ahmad ^1^, 700039113@uaeu.ac.ae, 0000-0002-7531-5264

Noura AlDhaheri ^1^, noura.aldhaheri@uaeu.ac.ae, 0000-0002-1384-2915

Mohd Saberi Mohamad ^1, 2, 3^, mohd.saberi@gmail.com, 0000-0002-1079-4559

Bassam R. Ali ^1*^, bassam.ali@uaeu.ac.ae, 0000-0003-1306-6618

1. Department of Genetics and Genomics, College of Medicine and Health Sciences, United Arab Emirates University, Al Ain, P.O. Box 15551, Sheikh Khalifa Bin Zayed Street, Al Maqam District, Abu Dhabi Emirate, United Arab Emirates.
2. Centre for Advanced Analytics, CoE for Artificial Intelligence, Faculty of Engineering & Technology, Multimedia University, Jalan Ayer Keroh Lama, Melaka, 75450 Bukit Beruang, Malaysia.
3. Department of Biosystems Engineering, Faculty of Agricultural Technology, Universitas Brawijaya, Malang, East Java, Jawa Timur 65145, Indonesia.
4. Institute For Data Innovation and Artificial Intelligence, Cranbourne East, VIC 3977, Australia.

***Correspondence:**

**Bassam R. Ali, PhD**

Department of Genetics and Genomics, College of Medicine and Health Sciences, United Arab Emirates University, United Arab Emirates.

E-mail address: bassam.ali@uaeu.ac.ae

Tel.: +971 - 505734708

ORCID: 0000-0003-1306-6618

**Supplementary Information**

**Gene List and Variant Curation**

To ensure that the study focused on genes of clear clinical and biological relevance to breast cancer, we curated a disease-specific gene panel using both literature and database evidence. First, a targeted literature review was performed across high-impact studies, reviews, and clinical reports describing hereditary and somatic breast cancer predisposition. Genes such as *BRCA1, BRCA2, TP53, PALB2, CHEK2, ATM*, and *CDH1* were consistently reported in multiple independent studies and therefore prioritized.

Second, the literature-derived list was cross-referenced with established variant repositories including ClinVar, COSMIC, TCGA, BRCA Exchange, and gnomAD. Only genes supported by both clinical and functional evidence were retained, thereby ensuring robust biological justification for inclusion.

Finally, clinical relevance was considered. The retained panel comprised genes with roles in DNA repair (*BRCA1/2, RAD51C*), cell-cycle regulation (*TP53, CHEK2*), and hereditary cancer syndromes (*ATM, CDH1*).

The curated panel included 107 genes, representing a comprehensive and clinically validated set of breast cancer-associated genes. Variant counts per gene are summarized in Supplementary Table S1, and per-gene distributions of benign and pathogenic variants are provided in Supplementary Table S2.

**Benign Balancing and Stratification**

Class imbalance was addressed by augmenting the benign variant set with population frequency data from gnomAD, following ACMG/AMP and ClinGen SVI guidelines. Variants exceeding the BA1 threshold (popmax FAF_95 ≥ 5%) or disease-specific maximum credible allele frequency cutoffs were classified as benign. Variants annotated as pathogenic/likely pathogenic in ClinVar or failing quality filters (non-PASS) were excluded.

Benign variants were sampled within each gene to approximately match pathogenic counts, while maintaining distribution across consequence categories (e.g., missense, splicing) and allele frequency bins. This ensured proportional gene-level representation: high-burden genes such as BRCA1, BRCA2, and TP53 retained higher counts, while low-burden genes were preserved at lower frequencies. Cross-validation was stratified by both gene and class, preventing bias and ensuring each fold reflected the true biological heterogeneity of breast cancer variant distributions.

The impact of balancing is detailed in Supplementary Table S2, which shows gene-level benign counts before and after augmentation. High-burden genes remain most represented, but proportional contribution is preserved across the panel.

**Benchmarking of Imputation Methods**

To evaluate strategies for handling missing data, we benchmarked KNN, MICE, and a Variational Autoencoder (VAE) under simulated missingness rates of 10%, 20%, and 30%. Performance was measured using both direct reconstruction errors (MAE, RMSE) and downstream classification metrics (AUC, weighted F1).

KNN consistently achieved the lowest reconstruction errors but showed fluctuating classifier performance across missingness rates. MICE achieved high AUC and F1 at low missingness (10%) but degraded sharply at 30%, with both MAE and RMSE increasing substantially. By contrast, VAE demonstrated the most stable performance, maintaining consistent AUC and F1 while avoiding the sharp degradation observed with MICE.

These results, shown in Supplementary Table S3 and Supplementary Figure 1, indicate that while KNN excels in raw imputation error, VAE provides the most robust downstream stability, making it better suited for high-dimensional genomic data where missingness is common and unevenly distributed.

**Feature Preprocessing and Selection**

**Correlation Filtering**

To reduce redundancy, we applied Pearson correlation filtering across thresholds ranging from |r| > 0.70 to 0.95. As shown in Supplementary Table S4, model performance (CV AUC) remained uniformly high (>0.999) across all thresholds. However, |r| > 0.90 provided the best trade-off, reducing multicollinearity while preserving predictive performance.

**Recursive Feature Elimination**

We next applied RFECV using ROC AUC as the scoring metric. The performance curve (Supplementary Figure 2) plateaued after approximately 15-20 features, confirming that only a subset was strictly required for maximal accuracy. To maintain interpretability while avoiding over-pruning, we retained 42 features at the |r| > 0.90 cutoff.

**Final Feature Set**

The retained features spanned complementary biological categories, including:

- Allele frequency metrics (AF, ALFA_Total_AN)
- Conservation scores (phyloP, phastCons, fitCons)
- Protein function predictors (SIFT, PolyPhen, BLOSUM62)
- Splicing predictors (SpliceAI ΔP scores)
- Meta-predictors/ensemble scores (ClinPred, REVEL, BayesDel, DANN)

The full feature set is provided in Supplementary Table S5. Together, these features capture orthogonal biological signals central to missense variant pathogenicity.

**Threshold Optimization**

To ensure optimal classification decisions, thresholds were optimized for each model using ROC and PR curve analyses. Two criteria were applied:

- Youden’s J statistic (maximizing TPR - FPR)
- F1-optimal cutoff (maximizing F1 from PR curves)

Cross-validation confirmed stability of selected thresholds. The Extra Trees classifier achieved the highest performance (AUC = 0.9997), with an optimal threshold of 0.20 (Supplementary Table S6). These results highlight the robustness of ensemble tree methods compared with linear and kernel-based models.

**Independent Validation Against ClinGen**

To test generalizability, we evaluated our Extra Trees model against an independent dataset curated from ClinGen Variant Curation Expert Panel resources. Predictions were compared with state-of-the-art meta-predictors, including MetaRNN, REVEL, and ClinPred.

As summarized in the main text (Table 7), Extra Trees achieved 99.1% accuracy and an MCC of 0.98, substantially outperforming other predictors, which ranged from 0.75-0.79 in accuracy. Only four misclassifications occurred with Extra Trees, compared with 74-96 for comparator methods.

Variant-level predictions are shown in Supplementary Table S7, where Extra Trees classifications were consistently aligned with curated clinical annotations. This external validation underscores both the robustness and translational potential of our approach.

**Supplementary Tables**

**Supplementary Table S1**. Genes list and variants count in each gene.

| **Gene** | **Variant count** | **Gene** | **Variant count** | **Gene** | **Variant count** |
| --- | --- | --- | --- | --- | --- |
| **TP53** | 3078 | **BRIP1** | 17 | **NRAS** | 5 |
| **BRCA2** | 2935 | **FANCM** | 17 | **PARP2** | 5 |
| **BRCA1** | 1261 | **FBXW7** | 16 | **POLD1** | 5 |
| **ERBB2** | 487 | **NBN** | 16 | **RAD51D** | 5 |
| **ATM** | 361 | **RB1** | 15 | **SETBP1** | 5 |
| **PTEN** | 298 | **ERBB3** | 14 | **ARPC1B** | 4 |
| **CDH1** | 296 | **RINT1** | 14 | **GEN1** | 4 |
| **MSH6** | 238 | **SLX4** | 14 | **PTPRF** | 4 |
| **MSH2** | 191 | **RET** | 13 | **SYNE2** | 4 |
| **CHEK2** | 153 | **IDH1** | 12 | **ALCAM** | 3 |
| **CTNNA1** | 116 | **MUTYH** | 12 | **ALK** | 3 |
| **PIK3CA** | 104 | **NTHL1** | 12 | **CDC73** | 3 |
| **NF1** | 86 | **PPM1D** | 12 | **FANCI** | 3 |
| **PMS1** | 81 | **RHOA** | 12 | **FGFR4** | 3 |
| **MLH1** | 63 | **BMPR1A** | 11 | **RECQL** | 3 |
| **BARD1** | 56 | **KRAS** | 11 | **SHBG** | 3 |
| **PALB2** | 53 | **POLE** | 11 | **VHL** | 3 |
| **BLM** | 50 | **EPCAM** | 10 | **AR** | 2 |
| **FANCD2** | 47 | **PALLD** | 10 | **FANCC** | 2 |
| **RAD51C** | 37 | **NF2** | 9 | **FLNC** | 2 |
| **ESR1** | 36 | **CDKN1A** | 8 | **MED12** | 2 |
| **SMARCA4** | 36 | **PDGFRA** | 8 | **RAD51** | 2 |
| **APC** | 35 | **WRAP53** | 8 | **SF3B1** | 2 |
| **GNAS** | 35 | **CASP8** | 7 | **CDKN2A** | 1 |
| **STK11** | 35 | **SDHA** | 7 | **CPS1** | 1 |
| **HRAS** | 33 | **ATXN7** | 6 | **DIS3L2** | 1 |
| **RAD51B** | 33 | **CACNA2D3** | 6 | **EIF2B5** | 1 |
| **MET** | 32 | **DCLRE1B** | 6 | **ERCC5** | 1 |
| **PMS2** | 30 | **FAM175A** | 6 | **FGFR3** | 1 |
| **EGFR** | 28 | **MTOR** | 6 | **GPC3** | 1 |
| **RAD50** | 25 | **PPP2R1A** | 6 | **JAK2** | 1 |
| **CDK12** | 24 | **SMAD4** | 6 | **KIT** | 1 |
| **MAP3K1** | 21 | **SUFU** | 6 | **MRE11A** | 1 |
| **TSC2** | 20 | **WRN** | 6 | **MYO7A** | 1 |
| **RAD54L** | 19 | **GALNT12** | 5 | **PTPN11** | 1 |
|  |  |  |  | **XRCC2** | 1 |

**Supplementary Table S2.** Representative gene-level counts before and after balancing, showing proportional augmentation of benign variants from gnomAD. High-burden genes (*BRCA1/2, TP53*) remain most represented, while lower-burden genes maintain proportional contribution.”

| **Gene** | **Benign variants before balancing** | **Benign variants after balancing** | **Gene** | **Benign variants before balancing** | **Benign variants after balancing** | **Gene** | **Benign variants before balancing** | **Benign variants after balancing** |
| --- | --- | --- | --- | --- | --- | --- | --- | --- |
| **ALCAM** | 0 | 1 | **FANCI** | 3 | 3 | **PMS1** | 81 | 81 |
| **ALK** | 0 | 1 | **FANCM** | 17 | 17 | **PMS2** | 20 | 21 |
| **APC** | 12 | 16 | **FBXW7** | 0 | 0 | **POLD1** | 0 | 0 |
| **AR** | 0 | 0 | **FGFR3** | 0 | 0 | **POLE** | 11 | 11 |
| **ARPC1B** | 4 | 4 | **FGFR4** | 0 | 0 | **PPM1D** | 12 | 12 |
| **ATM** | 147 | 199 | **FLNC** | 2 | 2 | **PPP2R1A** | 0 | 0 |
| **ATXN7** | 6 | 6 | **GALNT12** | 5 | 5 | **PTEN** | 47 | 100 |
| **BARD1** | 34 | 36 | **GEN1** | 4 | 4 | **PTPN11** | 0 | 0 |
| **BLM** | 41 | 42 | **GNAS** | 0 | 0 | **PTPRF** | 4 | 4 |
| **BMPR1A** | 6 | 8 | **GPC3** | 1 | 1 | **RAD50** | 15 | 15 |
| **BRCA1** | 834 | 839 | **HRAS** | 0 | 0 | **RAD51** | 0 | 0 |
| **BRCA2** | 1987 | 2093 | **IDH1** | 0 | 0 | **RAD51B** | 33 | 33 |
| **BRIP1** | 9 | 12 | **JAK2** | 0 | 0 | **RAD51C** | 20 | 20 |
| **CACNA2D3** | 6 | 6 | **KIT** | 0 | 0 | **RAD51D** | 5 | 5 |
| **CASP8** | 5 | 5 | **KRAS** | 0 | 0 | **RAD54L** | 18 | 18 |
| **CDC73** | 3 | 3 | **MAP3K1** | 21 | 21 | **RB1** | 15 | 15 |
| **CDH1** | 190 | 192 | **MED12** | 0 | 0 | **RECQL** | 3 | 3 |
| **CDK12** | 24 | 24 | **MET** | 26 | 28 | **RET** | 9 | 9 |
| **CDKN1A** | 8 | 8 | **MLH1** | 35 | 38 | **RHOA** | 0 | 0 |
| **CDKN2A** | 0 | 0 | **MRE11A** | 0 | 0 | **RINT1** | 14 | 14 |
| **CHEK2** | 110 | 112 | **MSH2** | 137 | 147 | **SDHA** | 7 | 7 |
| **CPS1** | 0 | 0 | **MSH6** | 198 | 202 | **SETBP1** | 5 | 5 |
| **CTNNA1** | 116 | 116 | **MTOR** | 0 | 0 | **SF3B1** | 0 | 0 |
| **DCLRE1B** | 6 | 6 | **MUTYH** | 12 | 12 | **SHBG** | 3 | 3 |
| **DIS3L2** | 0 | 0 | **MYO7A** | 0 | 0 | **SLX4** | 14 | 14 |

**Supplementary Table S2 (Continued).** Representative gene-level counts before and after balancing, showing proportional augmentation of benign variants from gnomAD. High-burden genes (*BRCA1/2, TP53*) remain most represented, while lower-burden genes maintain proportional contribution.”

| **Gene** | **Benign variants before balancing** | **Benign variants after balancing** | **Gene** | **Benign variants before balancing** | **Benign variants after balancing** | **Gene** | **Benign variants before balancing** | **Benign variants after balancing** |
| --- | --- | --- | --- | --- | --- | --- | --- | --- |
| **EGFR** | 22 | 24 | **NBN** | 11 | 13 | **SMAD4** | 0 | 0 |
| **EIF2B5** | 0 | 0 | **NF1** | 55 | 57 | **SMARCA4** | 35 | 35 |
| **EPCAM** | 10 | 10 | **NF2** | 8 | 8 | **STK11** | 11 | 15 |
| **ERBB2** | 30 | 32 | **NRAS** | 0 | 0 | **SUFU** | 6 | 6 |
| **ERBB3** | 3 | 3 | **NTHL1** | 12 | 12 | **SYNE2** | 3 | 3 |
| **ERCC5** | 0 | 0 | **PALB2** | 33 | 35 | **TP53** | 342 | 576 |
| **ESR1** | 0 | 0 | **PALLD** | 10 | 10 | **TSC2** | 15 | 16 |
| **FAM175A** | 6 | 6 | **PARP2** | 5 | 5 | **VHL** | 2 | 2 |
| **FANCC** | 0 | 0 | **PDGFRA** | 8 | 8 | **WRAP53** | 8 | 8 |
| **FANCD2** | 46 | 46 | **PIK3CA** | 3 | 3 | **WRN** | 3 | 3 |
|  |  |  |  |  |  | **XRCC2** | 0 | 0 |

**Supplementary Table S3.** Benchmarking of KNN, MICE, and VAE imputers under simulated missingness rates of 10%, 20%, and 30%. Performance was evaluated using mean AUC, weighted F1 score, RMSE, and MAE across repeated runs. KNN consistently achieved the lowest reconstruction error (RMSE and MAE), whereas VAE exhibited the most stable downstream classification performance across increasing missingness, highlighting its robustness for high-dimensional genomic variant data.

| **Method** | **Missingness Rate** | **Mean AUC** | **Mean Weighted F1** | **Mean RMSE** | **Mean MAE** |
| --- | --- | --- | --- | --- | --- |
| KNN | 0.1 | 0.820 | 0.7443 | 5261 | 49.7 |
| KNN | 0.2 | 0.821 | 0.7411 | 5475 | 62.5 |
| KNN | 0.3 | 0.818 | 0.7400 | 6461 | 80.6 |
| MICE | 0.1 | 0.825 | 0.7466 | 25832 | 1518.1 |
| MICE | 0.2 | 0.815 | 0.7426 | 29888 | 1824.5 |
| MICE | 0.3 | 0.819 | 0.7447 | 31254 | 1959.2 |
| VAE | 0.1 | 0.817 | 0.7420 | 26980 | 1538.7 |
| VAE | 0.2 | 0.814 | 0.7413 | 27630 | 1560.3 |
| VAE | 0.3 | 0.814 | 0.7397 | 28366 | 1551.5 |

**Supplementary Table S4**. Effect of Pearson correlation thresholds on feature count and model performance (5-fold CV AUC, Extra Trees).

| **abs_r_threshold** | **n_features** | **cv_AUC_mean** |
| --- | --- | --- |
| 0.7 | 15 | 0.999183 |
| 0.8 | 27 | 0.999066 |
| 0.85 | 35 | 0.999073 |
| 0.9 | 42 | 0.999588 |
| 0.95 | 63 | 0.99915 |

**Supplementary Table S5**. Final selected features and functional category.

| **Feature** | **Functional Category** |
| --- | --- |
| Allele | Variant descriptor |
| SYMBOL | Gene identifier |
| Gene | Gene identifier |
| EXON | Genomic location |
| HGVSc | Transcript notation |
| Amino_acids | Protein function |
| Codons | Protein function |
| REF_ALLELE | Variant descriptor |
| SWISSPROT | Protein annotation |
| SIFT | Protein function predictor |
| PolyPhen | Protein function predictor |
| AF | Allele frequency |
| MPC | Functional impact score |
| pTriplo | Dosage sensitivity |
| BLOSUM62 | Protein conservation |
| ALFA_Total_AN | Population frequency |
| BayesDel_addAF_rankscore | Ensemble pathogenicity score |
| DANN_rankscore | Ensemble pathogenicity score |
| GM12878_fitCons_rankscore | Conservation (cell-type specific) |
| H1-hESC_fitCons_rankscore | Conservation (cell-type specific) |
| HUVEC_fitCons_rankscore | Conservation (cell-type specific) |
| LIST-S2_score | Functional impact score |
| LRT_score | Evolutionary conservation |
| bStatistic_converted_rankscore | Constraint metric |
| integrated_fitCons_rankscore | Conservation (integrated) |
| phastCons17way_primate_rankscore | Conservation (primate) |
| phyloP17way_primate_rankscore | Conservation (primate) |
| Geno2MP_HPO_count | Phenotype association |
| SpliceAI_pred_DP_AG | Splicing predictor |
| SpliceAI_pred_DP_AL | Splicing predictor |
| SpliceAI_pred_DP_DG | Splicing predictor |
| SpliceAI_pred_DP_DL | Splicing predictor |

**Supplementary Table S6.** Benchmarking of classification models showing cross-validated AUC and optimal thresholds derived from ROC/PR analyses. Thresholds are reported using two criteria: Youden’s J statistic and F1-optimal cutoff. Results highlight the superior performance of ensemble tree methods (Extra Trees, Random Forest, XGBoost) compared with linear and kernel-based classifiers.

| **Classifier** | **AUC** | **Threshold_YoudenJ** | **Threshold_F1opt** |
| --- | --- | --- | --- |
| Random Forest | 0.999512 | 0.3 | 0.28 |
| XGBoost | 0.994106 | 0.197045 | 0.19171 |
| Logistic Regression | 0.793472 | 0.652398 | 0.408077 |
| SVM | 0.758409 | 0.648027 | 0.272668 |
| KNN | 0.922407 | 0.4 | 0.2 |
| Naive Bayes | 0.782055 | 0.827507 | 0.438509 |
| Decision Tree | 0.948363 | 1 | 0 |
| AdaBoost | 0.844351 | 0.492856 | 0.491103 |
| Extra Trees | 0.999687 | 0.29 | 0.22 |

**Supplementary Table S7.** Performance of nine machine learning classifiers trained with and without feature selection (seed = 42). Feature selection combined a Pearson correlation filter (|r| > 0.9) with RFECV to identify the optimal subset of features. The benchmarking study conducted earlier showed that very low feature counts reduce predictive performance, while increasing the number of features around or beyond the selected subset does not substantially change results. Accordingly, the chosen feature set represents a balance-simplifying computation and improving efficiency without sacrificing accuracy-while avoiding the performance drop observed with too few features. Metrics reported include AUC, F1, Precision, Recall, Specificity, Sensitivity, and MCC.

| **Model** | **AUC with FS** | **AUC no FS** | **F1 with FS** | **F1 no FS** | **Precision with FS** | **Precision no FS** | **Recall with FS** | **Recall no FS** | **Specificity with FS** | **Specificity no FS** | **Sensitivity with FS** | **Sensitivity no FS** | **MCC with FS** | **MCC no FS** |
| --- | --- | --- | --- | --- | --- | --- | --- | --- | --- | --- | --- | --- | --- | --- |
| **AdaBoost** | 0.8899 | 0.9016 | 0.8146 | 0.8066 | 0.8964 | 0.8875 | 0.7466 | 0.7392 | 0.9137 | 0.9063 | 0.7466 | 0.7392 | 0.6697 | 0.6548 |
| **SVM** | 0.7555 | 0.7551 | 0.7023 | 0.7017 | 0.6766 | 0.6763 | 0.73 | 0.7291 | 0.6511 | 0.6511 | 0.73 | 0.7291 | 0.3823 | 0.3813 |
| **Decision Tree** | 0.9513 | 0.9555 | 0.9494 | 0.9537 | 0.9891 | 0.994 | 0.9128 | 0.9164 | 0.9899 | 0.9945 | 0.9128 | 0.9164 | 0.9054 | 0.9137 |
| **KNN** | 0.9279 | 0.9301 | 0.8424 | 0.8486 | 0.94 | 0.9458 | 0.7631 | 0.7695 | 0.9513 | 0.9559 | 0.7631 | 0.7695 | 0.7274 | 0.7384 |
| **Extra Trees** | 0.9989 | 0.9993 | 0.9734 | 0.9814 | 0.9905 | 0.9953 | 0.9568 | 0.9679 | 0.9908 | 0.9954 | 0.9568 | 0.9679 | 0.9482 | 0.9636 |
| **XGBoost** | 0.9943 | 0.9969 | 0.9527 | 0.9659 | 0.993 | 0.9961 | 0.9155 | 0.9376 | 0.9936 | 0.9963 | 0.9155 | 0.9376 | 0.9119 | 0.9355 |
| **Random Forest** | 0.9987 | 0.999 | 0.9502 | 0.9635 | 0.992 | 0.9951 | 0.9118 | 0.9339 | 0.9927 | 0.9954 | 0.9118 | 0.9339 | 0.9075 | 0.9311 |
| **Naive Bayes** | 0.787 | 0.8091 | 0.7314 | 0.7519 | 0.7404 | 0.7301 | 0.7227 | 0.775 | 0.7466 | 0.7135 | 0.7227 | 0.775 | 0.4694 | 0.4894 |
| **Logistic Regression** | 0.8133 | 0.8234 | 0.7339 | 0.7609 | 0.7322 | 0.7465 | 0.7355 | 0.7759 | 0.7309 | 0.7365 | 0.7355 | 0.7759 | 0.4665 | 0.5128 |

Note: FS 🡪 Feature Selection

**Supplementary Figures**


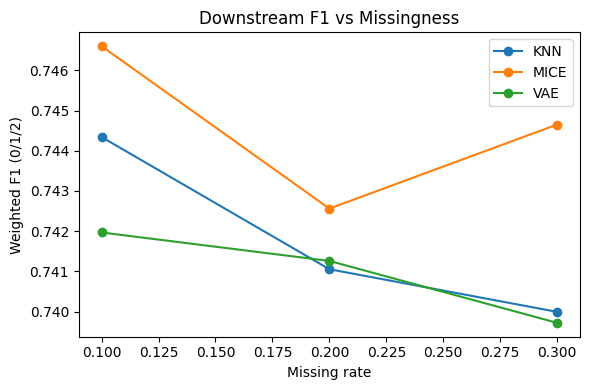

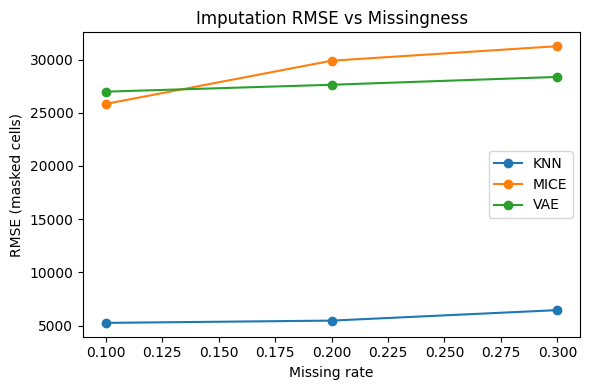

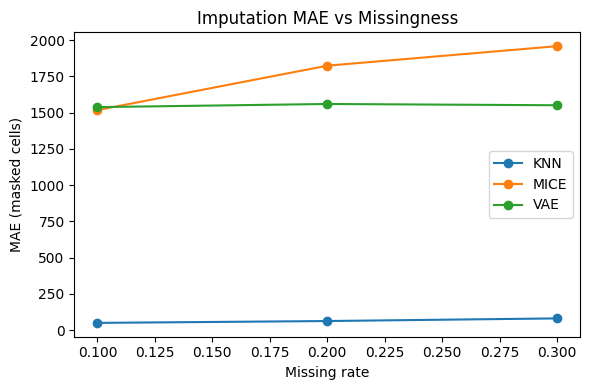

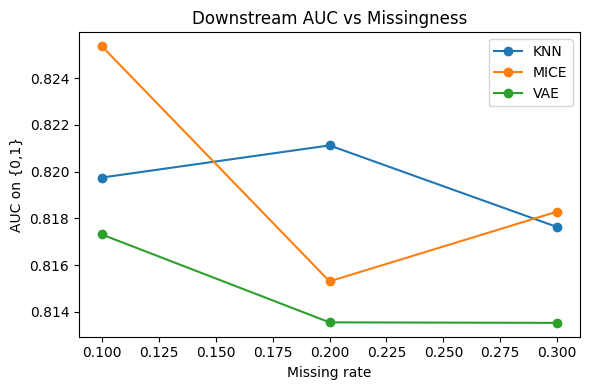

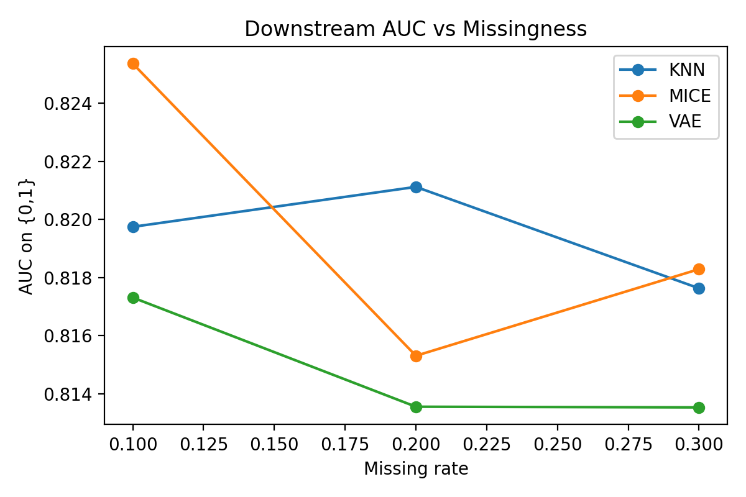


**Supplementary Figure 1. Performance of imputation strategies across increasing missingness levels.** Comparison of KNN, MICE, and VAE imputers at 10%, 20%, and 30% simulated missingness. Shown are the downstream classifier performance measured by mean AUC (top left, bottom left) and weighted F1 (bottom), and the imputation reconstruction error measured by RMSE (top right) and MAE (bottom right). MICE achieve the highest peak AUC and F1 at low missingness (10%) but degrades sharply as missingness increases. KNN exhibits fluctuating performance with increasing error. VAE demonstrates the most stable behaviour, maintaining consistent AUC, F1, RMSE, and MAE across missingness levels, indicating robustness under higher data loss. Error bars represent variation across cross-validation repeats.





**Supplementary Figure 2.** RFECV feature count benchmark. Cross-validated AUC is shown as a function of the number of features retained. Performance increased rapidly with the first few features and plateaued after ~15-20 features, indicating that only a subset was required for maximal accuracy. The final model retained 42 features, as determined by the Pearson correlation filter (|r| > 0.90), to balance predictive power with biological interpretability.
